# Right dose, right now: bedside, real-time, data-driven, and personalised antibiotic dosing in critically ill patients with sepsis or septic shock—a two-centre randomised clinical trial

**DOI:** 10.1186/s13054-022-04098-7

**Published:** 2022-09-05

**Authors:** Luca F. Roggeveen, Tingjie Guo, Lucas M. Fleuren, Ronald Driessen, Patrick Thoral, Reinier M. van Hest, Ron A. A. Mathot, Eleonora L. Swart, Harm-Jan de Grooth, Bas van den Bogaard, Armand R. J. Girbes, Rob J. Bosman, Paul W. G. Elbers

**Affiliations:** 1 Department of Intensive Care Medicine, Laboratory for Critical Care Computational Intelligence (LCCCI), Amsterdam Medical Data Science (AMDS), Amsterdam Cardiovascular Science (ACS), Amsterdam Institute for Infection and Immunity (AI&II), Amsterdam UMC, Vrije Universiteit, Amsterdam, The Netherlands; 2 Department of Pharmacy and Clinical Pharmacology, Amsterdam UMC, University of Amsterdam, Amsterdam, The Netherlands; 3 Division of Systems Biomedicine and Pharmacology, Leiden Academic Centre for Drug Research (LACDR), Leiden University, Leiden, The Netherlands; 4 Department of Intensive Care, OLVG Hospital, Amsterdam, The Netherlands

**Keywords:** Sepsis, Therapeutic drug monitoring, Pharmacokinetics, Clinical decision support

## Abstract

**Background:**

Adequate antibiotic dosing may improve outcomes in critically ill patients but is challenging due to altered and variable pharmacokinetics. To address this challenge, AutoKinetics was developed, a decision support system for bedside, real-time, data-driven and personalised antibiotic dosing. This study evaluates the feasibility, safety and efficacy of its clinical implementation.

**Methods:**

In this two-centre randomised clinical trial, critically ill patients with sepsis or septic shock were randomised to AutoKinetics dosing or standard dosing for four antibiotics: vancomycin, ciprofloxacin, meropenem, and ceftriaxone. Adult patients with a confirmed or suspected infection and either lactate > 2 mmol/L or vasopressor requirement were eligible for inclusion. The primary outcome was pharmacokinetic target attainment in the first 24 h after randomisation. Clinical endpoints included mortality, ICU length of stay and incidence of acute kidney injury.

**Results:**

After inclusion of 252 patients, the study was stopped early due to the COVID-19 pandemic. In the ciprofloxacin intervention group, the primary outcome was obtained in 69% compared to 3% in the control group (OR 62.5, CI 11.4–1173.78, *p* < 0.001). Furthermore, target attainment was faster (26 h, CI 18–42 h, *p* < 0.001) and better (65% increase, CI 49–84%, *p* < 0.001). For the other antibiotics, AutoKinetics dosing did not improve target attainment. Clinical endpoints were not significantly different. Importantly, higher dosing did not lead to increased mortality or renal failure.

**Conclusions:**

In critically ill patients, personalised dosing was feasible, safe and significantly improved target attainment for ciprofloxacin.

*Trial registration*: The trial was prospectively registered at Netherlands Trial Register (NTR), NL6501/NTR6689 on 25 August 2017 and at the European Clinical Trials Database (EudraCT), 2017-002478-37 on 6 November 2017.

**Supplementary Information:**

The online version contains supplementary material available at 10.1186/s13054-022-04098-7.

## Background

Mortality rates remain at 15–30% or even higher in critically ill patients with sepsis or septic shock [1–3]. Despite efforts to develop novel treatment strategies, antibiotics continue to be the cornerstone of sepsis treatment [4]. Their early and appropriate use has been associated with improved clinical outcomes [5, 6]. Appropriate use should also imply appropriate dosing. Overdosing may lead to toxicity associated with excess morbidity, while underdosing has been associated with increased antimicrobial resistance and poorer patient outcomes, including clinical cure and mortality [7–12].

However, achieving and maintaining adequate antibiotic levels is challenging, especially in the critically ill. These patients typically show markedly altered and rapidly changing pharmacokinetic (PK) profiles due to alterations in antibiotic clearance and volume of distribution. Contributing factors include hyperdynamic circulation, shifts in fluid balance, organ dysfunction and organ replacement therapies [7]. These factors vary greatly between patients, resulting in reported variations in antibiotic concentrations of over two orders of magnitude, but may also fluctuate extensively over time in any single patient [8]. Thus, adequate antibiotic dosing in the critically ill may be an important modifiable factor in optimising sepsis treatment. In line with the Precision Medicine Initiative [13] and the surviving sepsis campaign ranking personalised therapy as a top research priority [14], this calls for personalised antibiotic dosing in the critically ill.

Nevertheless, antibiotics typically continue to be dosed following standard regimens, even in the critically ill, perhaps related to inadequate PK knowledge among intensive care professionals [9]. But with the advent of electronic health records (EHRs), computerised decision support systems can now readily retrieve relevant individual patient data for model informed precision dosing [15]. Therefore, we developed AutoKinetics software to predict and graphically display patient specific antibiotic plasma concentrations in real time [16].

Providing direct and continuous individualised dosing advice directly to intensive care professionals at the bedside alleviates the need for manual data entry and guidance by pharmacists. Unlike therapeutic drug monitoring, feedback from antibiotic plasma levels is optional, and individual data-driven dosing advice is therefore available even before the first dose. To evaluate the feasibility, efficacy and safety of bedside, data-driven, and model informed precision dosing by AutoKinetics, we carried out a two-centre randomised clinical trial in critically ill patients with sepsis or septic shock.

## Methods

### Study design

The Right Dose Right Now study was an investigator-initiated, two-centre, randomised controlled, two-arm, paralleled, and non-blinded superiority trial conducted at Amsterdam UMC, location VUmc and OLVG, location East in Amsterdam, the Netherlands. Both are tertiary referral centres for intensive care medicine with medical and surgical patients. The full trial protocol was published while the trial was ongoing and before analyses of the results [17]. Additional details about the study protocol, including amendments, are detailed in the Additional file 1. Ethical approval was obtained in both centres (VUmc 2017.474, OLVG 18.011) and the trial was monitored by an independent clinical research bureau. This report follows the CONSORT guidelines [18].

### Patients and antibiotics

All adult intensive care patients were eligible for inclusion if they received antibiotics for a suspected or confirmed infection and had a suspected or measured serum lactate greater than two mmol/L or a requirement for vasopressor support in any dose.

Patients were eligible both at the start of and during an antibiotic course depending on when they fulfilled the inclusion criteria, specifically the lactate and vasopressor criteria. Patients could be prescribed multiple antibiotics using AutoKinetics but the clinical team could only include a patient for one antibiotic out of four antibiotics primarily used for the treatment of sepsis in the two centres: the β-lactam antibiotics ceftriaxone and meropenem, the fluoroquinolone ciprofloxacin, and the glycopeptide vancomycin. For all of these antibiotics, inadequate antibiotic exposure occurs with a frequency of up to 60% [8, 19, 20] and their appropriate dosing has been suggested to improve outcome [7]. The initial trial design also included the β-lactam cefotaxime, which could not be implemented due to a shortage at the national level and subsequent change in antibiotic treatment protocols for sepsis. There were no exclusion criteria. To avoid any delays in treatment, patients were included under deferred consent which was obtained from patients or their representatives within 48 h of randomisation.

### Randomisation and intervention

A randomisation module within AutoKinetics assigned patients to the control group or the intervention (AutoKinetics) group in a 1:1 allocation ratio with stratification by study centre, gender, and age group binned at 65 years using minimisation techniques [21]. The module rebalanced groups after patients were excluded. Patients remained allocated to their treatment group for the duration of their hospital admission. The trial was not blinded, and researchers and the treatment team were aware of treatment allocation. The pharmacokinetic endpoints, including primary endpoint of the study, were calculated using the same model based method for each patient relying on quantifiable plasma samples. The risk of bias in estimated treatment effects [22] is therefore low.

Integrated with two frequently used EHRs (MetaVision, iMDsoft, Tel Aviv, Israel and Epic (Epic Systems, Verona, WA, USA), AutoKinetics combines population PK models with relevant available EHR data. The user interface provides a graphical display of projected antibiotic plasma concentrations for all antibiotics and is available to the clinical team in real time (Additional file 1: Fig. S1). AutoKinetics calculates a dose advice for any predefined PK dosing target. Dosing advice was available at the start of an antibiotic course, arguably when target attainment may be most important [23] and when PK are most variable between critically ill patients [8]. Furthermore, AutoKinetics also provided dosing advice during an antibiotic course, even if the antibiotic course was started prior to inclusion.

In the AutoKinetics group, the individualised dose and dosing frequency recommendations were instantly available at the bedside for the primary antibiotic and the other three study antibiotics. Physicians were recommended to check AutoKinetics at least once per shift for dose advice, i.e. at least three times per day. The decision to follow the recommendations provided by AutoKinetics was at the discretion of the treating physician. In the control group, patients received antibiotics according to standard dosing regimens in each hospital, in line with international standards, as detailed in Additional file 1: Table S1. For feasibility reasons, the treatment team was not blinded to the dosing of antibiotics. The initiation and duration of the antibiotic course were left to the discretion of the clinical team. To ensure the appropriate use of and compliance with the AutoKinetics dose advice, intensive care unit (ICU) staffs, including residents, were trained at the start of their ICU rotation and quarterly thereafter for the duration of the trial. Physicians had the option to enter comments on their choice to accept or decline dosing recommendations and compliance was monitored.

Plasma sampling was scheduled for both treatment and control groups at least right after the first dose following randomisation, halfway through the first dosing interval, right before the second dose, and daily before a next dose for the following dosing intervals. For continuous infusions, multiple samples were scheduled with the first sample at least 1 h after a loading dose and at least once daily thereafter. Therapeutic drug monitoring with feedback on antibiotic plasma levels was applied for vancomycin as this was standard practice in the participating centres. All other plasma samples were stored at − 80 °C for up to one year, after which a liquid chromatography–mass spectrometry (LC–MS) analysis method was used to determine total plasma concentration for the four study antibiotics. Samples were blinded and analysed by the pharmacy laboratory, and results were reported by study ID to the research team only.

### AutoKinetics dosing strategy

For each antibiotic, best performing PK models were selected, validated, calibrated and implemented in AutoKinetics, as published previously [16]. Vancomycin and ciprofloxacin are time- and concentration-dependent antibiotics, and their efficacy is related to the area under the concentration versus time curve (AUC) relative to the pathogen minimum inhibitory concentration (MIC). Meropenem and ceftriaxone are time-dependent antibiotics where time above a concentration relative to the MIC is best associated with their efficacy. As true MIC values were not routinely available from the electronic health record, a surrogate value of 1 mg/L was used, based on EUCAST clinical breakpoints to avoid delays and allow for empirical therapy [24]. Dosing targets for each antibiotic were based on clinical and preclinical studies as previously reviewed and focus on the prevention of underdosing [7]. Detailed dosing strategies and rationale may be found in the Additional file 1 and Additional file 1: Table S1.

### Outcomes

All outcome definitions were prespecified and described in detail in the published study protocol [17]. The primary endpoint was PK target attainment (TA), during the first 24 h following randomisation for the primary antibiotic for which the patient was randomised. The primary endpoint was prespecified at 75% of the dosing target, a conservative endpoint [7], as detailed in Additional file 1: Table S1. Secondary PK outcomes were time to PK target attainment (TTA) and the fraction of days of the entire antibiotic course up to ICU discharge during which the primary outcome was reached (%-TA). All PK outcomes were assessed for each antibiotic separately.

Secondary clinical outcomes included mortality at ICU discharge, hospital discharge, day 28 and 6 months as well as ICU- and hospital length of stay, the delta Sequential Organ Failure Assessment (SOFA) score [25] between baseline on the day of randomisation and 96 h after, and incidence of acute kidney injury (AKI).

Finally, a predefined safety analysis was performed for subgroups based on the cumulative dose administered in the first 24 h after randomisation compared to the cumulative daily standard dose, unadjusted for kidney function: low dosing (< 50% of standard dose), normal dosing (dose within 50% and 200%) and high dosing (> 200%). For this safety analysis, clinical outcomes were assessed regardless of the choice of primary antibiotic, focusing on mortality and renal failure.

### Pharmacokinetic and statistical analysis

The full analysis plan including sample size calculations was published previously as part of the study protocol [17]. Target attainment was derived from measured antibiotic plasma concentrations and maximum a posteriori Bayesian estimation using the AutoKinetics population PK models. Concentration data were aggregated in one-hour time steps to calculate the AUCs using the trapezoidal method.

For all PK outcomes, an intention-to-treat analysis was performed. Mixed effects modelling was used to account for potentially modifying effects from the stratification factors gender, age and treatment centre. A time-to-event analysis was performed to generate Kaplan–Meier survival plots using a Cox regression model. Between-group comparisons of clinical outcomes were assessed using the two-group Chi-square test or Kruskal–Wallis rank sum test where applicable. The DALI study [8] showed that 60% of patients did not reach the PK targets. We performed a power analysis (alpha 0.05, 1-beta 0.80) which showed a required sample size of 42 patients per group, per antibiotic, based on a clinically relevant reduction from 60 to 30% of failure to attain PK targets.

We performed an exploratory PK target analysis to evaluate the effect of AutoKinetics dosing for different PK targets. We calculated the probability of target attainment for multiple targets over a range of MIC values to assess the effect of AutoKinetics dosing in more detail. As a post hoc analysis, PK model accuracy and precision were assessed as the absolute prediction error between the observed and predicted plasma concentrations, and relative prediction error, respectively. Finally, we performed a post hoc analysis to evaluate target attainment for vancomycin after initiation of therapeutic drug monitoring (TDM) to assess the effect of bedside dosing advice independent from model performance, as Bayesian optimisation for TDM corrects for model inaccuracy.

Statistical analyses were carried out in R (version 4.0.3; www.R-project.org) and Python (version 3.7; www.python.org). Pharmacokinetic analyses were performed in NONMEM (version 7.5; ICON Development Solutions, MD, USA). Where applicable and as such denoted, secondary endpoints have been adjusted for multiplicity and their unadjusted *p* values should be interpreted as exploratory only.

## Results

### Patients and data

**Fig. 1:**
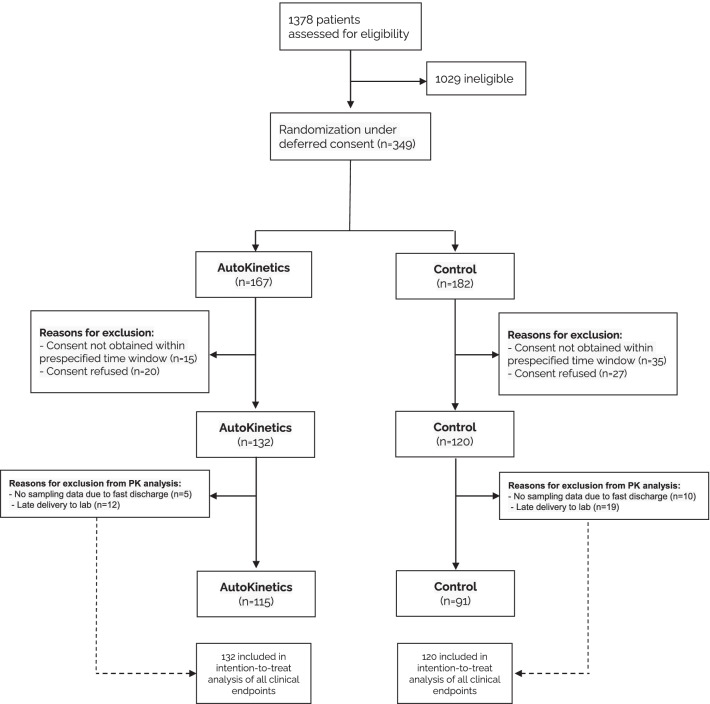
Flow diagram of enrolment and randomisation of patients. Patient flow for the trial. Patients were randomised under deferred consent and exclusions therefore occur after the start of the intervention. The randomisation software rebalanced groups after exclusions to maintain equal stratified group sizes. PK = pharmacokinetic

From 2 February 2018 until 20 March 2020, a total of 349 patients were enrolled in both centres. Informed consent was obtained for 252 patients. Of these patients, 132 were randomised to the AutoKinetics group and 120 to the control group. The trial was stopped early due to the COVID-19 pandemic. As a result, recruitment was completed for ceftriaxone and ciprofloxacin but not for vancomycin and meropenem. Patient flow and reasons for exclusion after randomisation can be found in Fig. 1.

Overall, the two groups were balanced with regard to their baseline characteristics (Table 1). At randomisation, 44% of patients fulfilled the sepsis-3 criteria for septic shock [1]. Most patients were randomised in the early (median 4.8, interquartile range 0.9–26.5) hours of their ICU admission and started their antibiotic therapy immediately thereafter.

**Table 1:** Baseline characteristics of trial patients

	AutoKinetics (132)	Control (120)
Age, mean (IQR)—years	68 (60–75)	67 (56–74)
Male, n (%)	90 (68.2)	82 (68.3)
Body Mass Index, median (IQR)—kg/m^2^	25.5 (23.4–29.7)	26.0 (22.5–29.3)
Weight, median (IQR)—kg	80 (70–91)	80 (70–92)
SOFA score on day of randomisation, median (IQR)	10.0 (7.0–13.0)	10.0 (7.0–12.0)
Leukocytes at randomisation (*10^9), median (IQR)	14.7 (8.9–22.1)	13.3 (8.4–18.7)
C-reactive protein at randomisation, median (IQR)	142.0 (71.0–296.2)	165.0 (65.0–283.0)
Creatinine at randomisation, median (IQR)	117.5 (78.2–165.5)	126.0 (77.0–197.5)
Septic shock^a^ at randomisation, n (%)	48 (36.4)	63 (52.5)
KDIGO stage at randomisation, n (%)		
0	80 (60.6)	68 (56.7)
1	32 (24.2)	28 (23.3)
2	9 (6.8)	15 (12.5)
3	11 (8.3)	9 (7.5)
Comorbidities, n (%)		
Diabetes	18 (21.4)	11 (14.3)
Renal insufficiency	16 (12.1)	15 (12.5)
Cardiovascular insufficiency	9 (6.8)	6 (5.0)
Malignancy	24 (18.2)	22 (18.4)
Immunological insufficiency	29 (22.0)	27 (22.5)
Primary affected organ system upon admission, n (%)		
Cardiovascular	71 (54.2)	99 (55.0)
Respiratory	31 (23.7)	32 (26.7)
Gastrointestinal	18 (13.7)	16 (14.4)
Trauma	5 (3.8)	3 (2.5)
Neurologic	3 (2.3)	1 (0.8)
Other	5 (3.8)	2 (1.6)
Admission characteristics		
Time from ICU admission until randomisation, median (IQR)—hours	3.4 (0.9–23.6)	5.5 (0.8–21.1)
Antibiotic course initiation after randomisation, n (%)	74 (56.9)	76 (63.9)
Primary antibiotic, n (%)		
Vancomycin	16 (12.1)	16 (13.3)
Ciprofloxacin	49 (37.1)	43 (35.8)
Meropenem	24 (18.2)	20 (16.7)
Ceftriaxone	43 (32.6)	41 (34.2)
Coadministered study antibiotic, n (%)		
Vancomycin	29 (22.0)	26 (21.7)
Ciprofloxacin	33 (25.0)	38 (31.7)
Meropenem	13 (9.8)	5 (4.2)
Ceftriaxone	31 (23.5)	28 (23.3)

SOFA, Sequential Organ Failure Assessment; CRP, C-Reactive Protein; IQR, Interquartile Range; KDIGO, Kidney Disease Improving Global Outcomes

^a^Septic shock is defined using the Sepsis-3 criteria: Sepsis with a lactate > 2 and use of vasopressors

Due to delays in plasma sample storage, transport and determination, samples could not be analysed for some patients and were discarded. Antibiotic concentration data for the calculation of the primary outcome were available for 85% of the ceftriaxone patients, 78% of the ciprofloxacin patients, 90% of the meropenem patients and 76% of the vancomycin patients. Pharmacokinetic outcomes are reported for these patients while clinical outcomes are reported for all patients regardless of plasma sample availability. Given blinded analysis and reporting, missingness is likely to have occurred at random.

### Pharmacokinetic outcomes

For ciprofloxacin, the primary outcome of target attainment in the first 24 h was reached in 69% of patients in the AutoKinetics group compared to 3% in the control group (OR 62.5 CI 11.4–1173.8, *p* < 0.001). For ceftriaxone, vancomycin, and meropenem, there were no statistically significant differences in PK target attainment (Table 2). The cumulative dose for the primary antibiotic given in the first 24 h after randomisation is shown in Additional file 1: Fig. S2. Additional details about the dosing regimens and measured plasma concentrations are available in Additional file 1: Figs. S3 and S4. A significant difference was observed for ciprofloxacin with a daily median dose of 2600 mg (IQR 2000–3000 mg) in the AutoKinetics group versus 1000 mg (IQR 800–1200 mg) in the control group.

**Table 2:** Pharmacokinetic primary and secondary outcomes

	AutoKinetics N = 115	Control N = 91	delta estimate (confidence interval)	Odds ratio (confidence interval)	*p* value
*Target attainment within 24 h, n / N (%)*					
Vancomycin	12/12 (100%)	13/14 (93%)		Inf (0.02 to inf)^x^	1^x^
Ciprofloxacin	29/42 (69%)	1/29 (3%)		62.5 (11.4 to 1173.78)^a^	< 0.001
Meropenem	12/19 (63%)	12/15 (80%)		0.42 (0.07 to 1.95)^a^	0.291
Ceftriaxone	37/39 (95%)	31/32 (97%)		0.60 (0.03 to 6.51)^a^	0.679
*Fraction of days with target attainment (FTA)—median (IQR)*					
Vancomycin	1 (1 to 1)	1 (0.93 to 1)	0.01 (− 0.18 to 0.58)^b^		0.42
Ciprofloxacin	1 (0.5 to 1)	0 (0 to 0)	0.65 (0.42 to 0.88)^b^		< 0.001
Meropenem	1 (0 to 1)	1 (0.75 to 1)	− 0.15 (− 0.45 to 0.18)^b^		0.413
Ceftriaxone	1 (1 to 1)	1 (1 to 1)	− 0.08 (− 0.08 to 0.01)^b^		0.889
*Time in hours to target attainment (TTA)—median (IQR)*					
Vancomycin	7 (0 to 8.3)	10 (6.1 to 13.5)	− 3.23 (− 7.38 to 0.92)^c^		0.141
Ciprofloxacin	17.7 (14.1 to 23.1)	41.2 (33.0 to 54.4)	− 26.00 (− 32.45 to − 18.71)^c^		< 0.001
Meropenem	4.97 (0 to 20.0)	0.08 (0 to 18.6)	2.87 (− 8.26 to 14.42)^c^		0.618
Ceftriaxone	14.9 (6.4 to 18.4)	18.2 (10.2 to 18.9)	− 2.10 (− 6.06 to 1.69)^c^		0.276

The target was defined as 75%-T0-24 > 4⋅MIC for ceftriaxone and meropenem, where 75%T0-24 denotes 75% of the time. For vancomycin, target was defined as AUC0-24/MIC > 300 and for ciprofloxacin as AUC0-24/MIC > 94, which is also 75% of the target used for AutoKinetics dose advice

IQR, Interquartile Range; SD, standard deviation

^x^Fisher’s exact test was used for null-hypothesis testing and confidence interval calculation due to the 100% target attainment in the antibiotic group rather than a generalised linear mixed model

^a^Odds ratio with confidence interval around the odds, calculated using a generalised linear mixed model on a binomial distribution

^b^Difference in fraction of days with target attainment with confidence interval, calculated based on a linear mixed model

^c^Difference in hours to target attainment with confidence interval, calculated based on a linear mixed model. A negative TTA difference indicates a reduction in time to target attainment for the AutoKinetics group compared to control

**Fig. 2:**
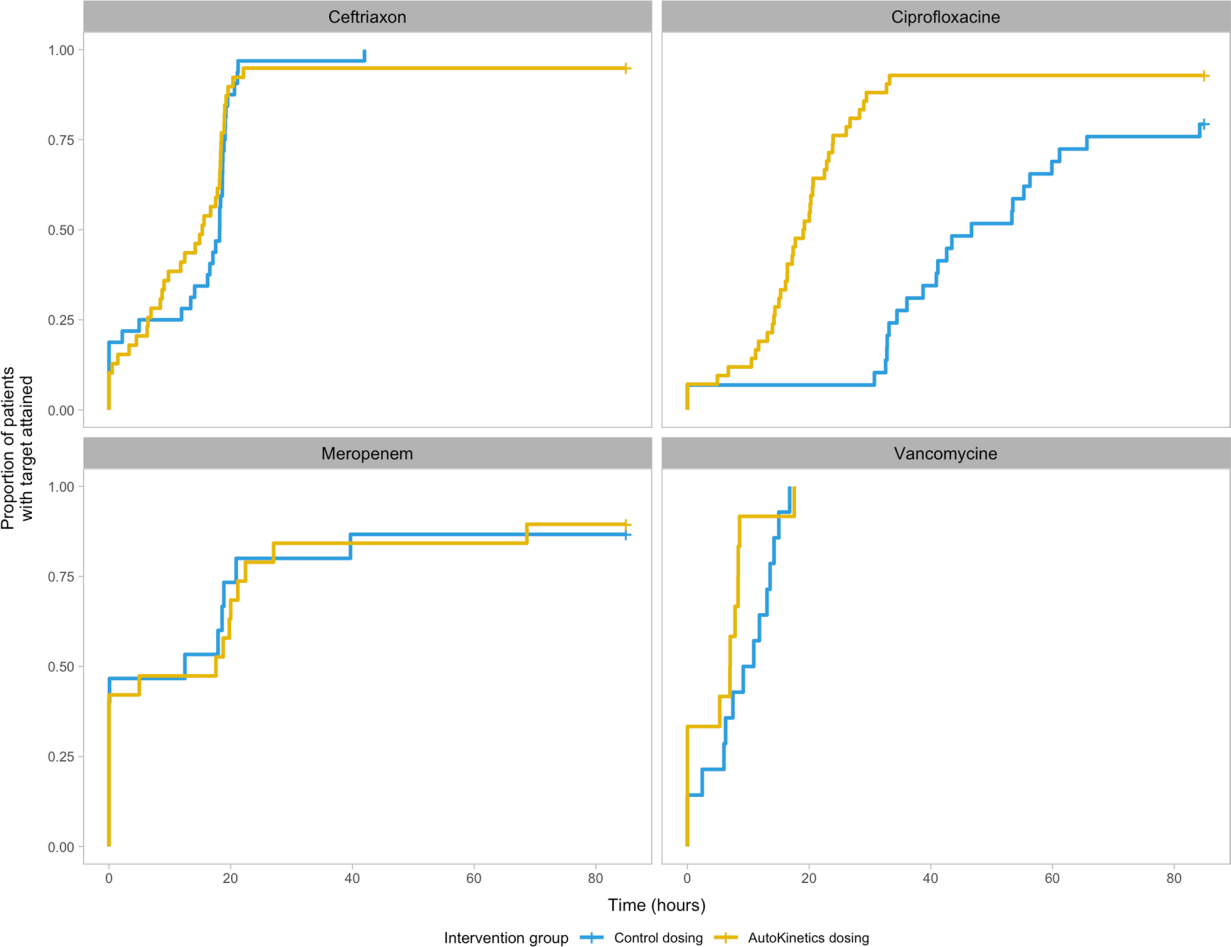
Time to target attainment survival analysis. A survival analysis of the time until primary target attainment for each antibiotic. The *Y* axis denotes the proportion of patients (up to 1) that have reached their PK target. The *X* axis denotes the time in hours it takes to reach the primary target. Lines that stop prematurely have reached a proportion of 1. At hour zero, some patients are already on target because they were randomised after their first antibiotic dose. For some antibiotics, target attainment can be reached almost directly after the first dose as can be seen in the case of meropenem, while for others, most notably ciprofloxacin, target attainment requires several hours up to days

The secondary PK outcomes are shown in Table 2. Additionally, the survival analysis for the TTA is shown in Fig. 2. For ciprofloxacin, TTA was significantly shorter in the AutoKinetics group with a median time reduction of 26 h (CI 18–42 h, *p* value < 0.001). There was no significant difference in TTA for ceftriaxone, vancomycin, and meropenem. Patients in the AutoKinetics group showed a 65% (CI 42–88%, *p* value < 0.001) increase in days on target for the full antibiotic course for ciprofloxacin, but not for the other antibiotics (Table 2).

### Clinical outcomes

No significant differences were observed between AutoKinetics and the control group for the secondary clinical endpoints (Table 3). Importantly, no increase in SOFA score, kidney failure, AKI severity or initiation of Continuous Veno-Venous Hemofiltration (CVVH) was found in the AutoKinetics group. The point estimates for mortality and renal outcomes favoured the AutoKinetics group, although these differences did not reach statistical significance.

**Table 3:** Clinical safety outcomes

	AutoKinetics (132)	Control (120)	*p* value
ICU mortality, n (%)	45 (34.1)	44 (36.7)	0.768^a^
Hospital mortality, n (%)	45 (34.1)	47 (39.2)	0.481^a^
28-day mortality, n (%)	45 (34.1)	48 (40.0)	0.401^a^
6-month mortality, n (%)	56 (42.4)	59 (49.2)	0.344^a^
New onset AKI, n (%)	44 (33.3)	50 (41.7)	0.217^a^
Highest KDIGO stage, n (%)			0.081^a^
0	30 (22.7)	15 (12.5)	
1	11 (8.3)	19 (15.8)	
2	47 (35.6)	44 (36.7)	
3	44 (33.3)	42 (35.0)	
Received CVVH, n (%)	18 (13.6)	19 (15.8)	0.754
Days free from CVVH, mean (SD)	10.0 (18.8)	8.5 (11.4)	0.856^b^
SOFA score at 96 h, median (IQR)	10.0 (6.0 to 15.2)	10.0 (5.0 to 19.2)	0.919^b^
Delta SOFA score at 96 h, median (IQR)	0.0 (− 3.0 to 4.0)	0.0 (− 2.0 to 4.0)	0.238^b^
Hospital LOS, median (IQR)	14.0 (4.6 to 30.2)	12.2 (3.4 to 28.4)	0.437^b^
ICU LOS, median (IQR)	3.8 (1.6 to 11.7)	3.6 (1.0 to 11.0)	0.594^b^

AKI, Acute Kidney Injury; CVVH, Continuous Veno-Venous Hemofiltration; KDIGO, Kidney Disease: Improving Global Outcomes; LOS, Length of stay; SOFA, Sequential Organ Failure Assessment; IQR, interquartile range

^a^Chi-squared

^b^Kruskal–Wallis

The subgroup analysis for high, low and normal dosing is shown in Table 4. No differences in clinical outcomes were observed between these groups. Importantly, exposure to high dosing by AutoKinetics did not lead to an increase in new kidney failure, an increase in AKI severity or use of renal replacement therapy. Rather, trends were favourable for these outcomes, as well as delta SOFA scores, in the AutoKinetics group.

**Table 4:** Safety dosing group analysis

	AutoKinetics high dosing group (36)	AutoKinetics low dosing group (24)	AutoKinetics Normal dosing group (72)	Control (120)	*p* value (adjusted)^a^
ICU mortality, n (%)	11 (30.6)	7 (29.2)	27 (37.5)	44 (36.7)	0.801 (0.998)
Hospital mortality, n (%)	11 (30.6)	9 (37.5)	27 (37.5)	47 (39.2)	0.667 (0.997)
28-day mortality, n (%)	11 (30.6)	7 (29.2)	27 (37.5)	48 (40.0)	06.27 (0.997)
6-month mortality, n (%)	13 (36.1)	10 (41.7)	33 (45.8)	59 (49.2)	0.557 (0.997)
New onset AKI, n (%)	16 (44.4)	3 (12.5)	25 (34.7)	50 (41.7)	0.041 (0.416)
Highest KDIGO stage, n (%)					0.119 (0.752)
0	10 (27.8)	5 (20.8)	15 (20.8)	15 (12.5)	
1	4 (11.1)	3 (5.6)	4 (5.6)	19 (15.8)	
2	16 (44.4)	6 (25.0)	25 (34.7)	44 (36.7)	
3	6 (16.7)	10 (41.7)	28 (38.9)	42 (35.0)	
Received CVVH, n (%)	2 (5.6)	5 (20.8)	11 (15.3)	19 (15.8)	0.352 (0.980)
Days free from CVVH, median (IQR)	3.0 (2.0 to 9.2)	3.0 (1.0 to 10.5)	4.5 (2.0 to 10.0)	4.0 (2.0 to 10.0)	0.795 (0.998)
SOFA score at 96 h, median (IQR)	9.0 (5.0 to 12.2)	13.5 (10.0 to 24.0)	9.0 (5.8 to 14.2)	10.0 (5.9 to 19.2)	0.075 (0.607)
Delta SOFA score at 96 h, median (IQR)	− 1.0 (− 3.0 to 4.0)	0.0 (− 1.5 to 4.0)	0.0 (− 3.0 to 3.2)	0.0 (− 2.0 to 4.0)	0.523 (0.997)
Hospital LOS, median (IQR)	12.7 (5.4 to 26.5)	29.3 (1.1 to 37.4)	14.0 (6.5 to 29.3)	12.2 (3.4 to 28.4)	0.828 (0.998)
ICU LOS, median (IQR)	2.3 (1.8 to 7.0)	3.5 (1.1 to 11.2)	4.2 (1.6 to 11.7)	3.6 (1.0 to 11.0)	0.792 (0.998)
Coadministered study antibiotic, n (%)					
Vancomycin	6 (17.6)	4 (15.4)	19 (26.4)	26 (21.7)	
Ciprofloxacin	4 (11.8)	10 (38.5)	19 (26.4)	38 (31.7)	
Meropenem	2 (5.9)	2 (7.7)	9 (12.5)	5 (4.2)	
Ceftriaxone	12 (35.3)	2 (7.7)	17 (23.6)	28 (23.3)	

Safety groups (< 50% & > 200% of normal, unadjusted for kidney function, daily dose) are based on the cumulative dose in the first 24 h after randomisation for the primary antibiotic

AKI, Acute Kidney Injury; CVVH, Continuous Veno-Venous Hemofiltration; KDIGO, Kidney Disease: Improving Global Outcomes; LOS, Length of stay; SOFA, Sequential Organ Failure Assessment; IQR, interquartile range

^a^p value was adjusted for multiplicity using the Holm-Sidak method

### Compliance

*Less than* 2% of all dosing recommendations were rejected by the clinical team. However, physicians were required to manually enter or alter the antibiotic order in the EHR system and deviations from recommendations may still have occurred, although this is unlikely given the wide dose distribution observed.

### Pharmacokinetic exploratory analysis

We calculated the probability of target attainment for the primary outcome for a range of MIC cut-offs and for both the PK outcome and the PK dosing target, see Additional file 1: Fig. S5. Notably, for ciprofloxacin and vancomycin, AutoKinetics dosing led to higher target attainment for a wide range of PK targets in the first 24 h after randomisation, but not for meropenem and ceftriaxone.

We assessed the effect of AutoKinetics in combination with TDM. AutoKinetics resulted in higher average AUCs for the entire antibiotic course and a trend towards more accurate target attainment after TDM, showing a reduction in overdosing compared to clinical practice (Additional file 1: Fig. S6). Secondly, we analysed the accuracy and precision of the PK models for the AutoKinetics population. For meropenem, we found a higher risk of underdosing with the implemented AutoKinetics model, while for ceftriaxone, we found a higher risk of overdosing (Additional file 1: Table S2).

## Discussion

This study demonstrates that computerised decision support for model based, continuous, real-time antibiotic dosing advice at the intensive care bedside for critically ill patients with sepsis or septic shock is feasible and safe.

AutoKinetics significantly improved PK target attainment in patients with sepsis or septic shock for ciprofloxacin and more than halved the time to adequate exposure. Importantly, as shown in our exploratory analyses, target attainment for ciprofloxacin standard dosing remains minimal even for lower PK targets. Current treatment protocols on ciprofloxacin dosing may therefore be considered inadequate, especially in areas with high antimicrobial resistance [23].

For ceftriaxone, meropenem, and vancomycin, AutoKinetics dosing did not lead to better PK target attainment. Potential explanations for different results for the different classes of antibiotics include the performance of the models used, the target used for dosing and evaluation, and the timing of our intervention relative to initiation of antibiotic therapy.

We observed no differences in the clinical endpoints between the two groups. Overall, mortality and renal outcomes were similar between groups and comparable to the literature [26]. In addition, the prespecified dosing group safety analysis showed that even large dose adjustments by AutoKinetics are clinically safe. This is especially important for ciprofloxacin given that the exposure has more than doubled compared to the control group, partly due to a high loading dose, while the AKI scores trend favourable for the AutoKinetics high dosing group.

For an increasing number of antibiotics, pharmacist supported therapeutic drug monitoring programmes have been implemented in hospitals and their ICUs. This typically leads to dose adjustments every 24 to 72 h. AutoKinetics incorporates antibiotic plasma level feedback, if available, but without the need for human interpretation and can thus provide continuous dosing support to the clinician. Our exploratory analysis for vancomycin, in which both groups benefited from plasma level feedback, revealed that AutoKinetics resulted in less variation in exposure without a decrease in PK target attainment. This may be relevant as overall exposure is a prominent risk factor for vancomycin toxicity [27].

It is currently insufficiently understood what exact PK targets should be targeted to improve clinical outcomes and total plasma concentration targets may not necessarily be associated with antimicrobial efficacy at the site of infection. Furthermore, it is currently unclear if individual MIC measurements are suited for precision dosing as these are error prone [28] and frequently unavailable at initiation of treatment. Therefore, the PK targets in this trial were based on EUCAST epidemiological cut-offs based on combinations of prevalent pathogens and the antibiotics studied. In addition, safety margins were applied to account for MIC variation to prevent underdosing. Different MIC targets can be incorporated depending on the clinical situation and regional microbial resistance. AutoKinetics is designed to account for any desired PK target and is compatible with any compartment PK model. This provides the flexibility to function in a range of clinical settings.

This trial also has limitations. In a post hoc analysis, we found high prediction errors for the PK models for ceftriaxone and meropenem, which may have led to overdosing and underdosing for these antibiotics, respectively. Thus, better PK models need to be developed for use in clinical practice.

We did not use free fraction antibiotic concentration for the dosing target which may be a better PK target than total (bound and unbound) plasma concentration. This is especially important for ceftriaxone which is known to have a high variability in protein binding and for which unbound plasma concentration may be better associated with antimicrobial efficacy at the site of infection [29].

Furthermore, patients could be included in the trial at the beginning of their antibiotic course but also at a later stage. The maximum potential benefit of AutoKinetics may be limited to those patients who were included from the start of their antibiotic course. Similarly, patients could have received multiple and concurrent antibiotic courses and it is unclear which antibiotic course was most clinically beneficial or effective. As prespecified, an analysis has been planned—that also includes all secondary antibiotic courses—to evaluate the relationship between different PK targets and clinical and microbiological cure.

Lastly, due to the COVID-19 pandemic, the trial was stopped prematurely at about 75% of intended inclusions. A larger sample size may have yielded more conclusive results for the vancomycin and meropenem group and could have strengthened the signal for clinical benefit for AutoKinetics. Future studies to investigate the benefit of personalised dosing on clinical outcomes should therefore be encouraged.

## Conclusions

We found that AutoKinetics, an EHR integrated computerised decision support system for model informed precision dosing for antibiotics made available directly to intensive care professionals at the bedside, is feasible, safe and can be used to improve antibiotic dosing for patients with sepsis or septic shock.

## Supplementary Information

**Additional file 1**. Electronic supplementary material.

## Data Availability

Deidentified participant data with a data dictionary will be made accessible with investigator support upon request within the limitations of applicable laws and regulations.

## Abbreviations

PK: Pharmacokinetic
EHR: Electronic Health Record
ICU: Intensive Care Unit
LC–MS: Liquid Chromatography–Mass Spectrometry
AUC: Area Under the Curve
MIC: Minimal Inhibitory Concentration
TA: Target Attainment
TTA: Time to Target Attainment
AKI: Acute Kidney Injury
TDM: Therapeutic Drug Monitoring
CVVH: Continuous Veno-Venous Hemofiltration
