# Supplementary material for: Right dose, right now: bedside, real-time, data-driven, and personalised antibiotic dosing in critically ill patients with sepsis or septic shock—a two-centre randomised clinical trial
Source: Crit Care. 2022 Sep 5;26:265. doi: 10.1186/s13054-022-04098-7 (PMC9443636; doi:10.1186/s13054-022-04098-7)
Supplement: Supplementary file 1 — Additional file 1. Electronic supplementary material. [file 13054_2022_4098_MOESM1_ESM.docx]

Right dose, right now: bedside, real-time, data-driven, and personalised antibiotic dosing in critically ill patients with sepsis or septic shock - a two-centre randomised clinical trial

Supplementary material

Table of contents

Standard regimen dosing protocols for study participants 3

Clinical breakpoints for minimal inhibitory concentration 3

Study protocol and important changes after trial commencement 3

Supplementary material references 4

Supplementary Figure 1. AutoKinetics interface 5

Supplementary Figure 2. Dose distribution in the first 24 hours after randomisation 6

Supplementary Figure 3. Individual dose distribution in the first 7 days after randomisation 7

Supplementary Figure 4. Measured plasma concentration for the primary antibiotic courses 8

Supplementary Figure 5. Probability of target attainment for the first 24 hours after randomisation 9

Supplementary Figure 6. Average AUC_24_ for the entire antibiotic course versus that after TDM initiation for vancomycin 10

Supplementary Table 1. Antibiotic dosing schedule for routine clinical practice 11

Supplementary Table 2. Prediction error of AutoKinetics pharmacokinetic models 12

# Standard regimen dosing protocols for study participants

Antibiotic dosing in Amsterdam UMC is based on the Dutch institution ‘Stichting Werkgroep Antibiotica Beleid’ (SWAB) and is publicly available at <https://amsterdamumc.adult.swabid.nl/>. These publicly available protocols may undergo future updating. For reference, we have therefore specified the standard dosing practice for the 4 antibiotics in the trial which were routine practice for the duration of this trial. These are shown in Supplementary Table 1.

# Clinical breakpoints for minimal inhibitory concentration

Dosing targets were based on EUCAST clinical breakpoints in 2018, available at the start of the trial [1]. Notably, for vancomycin, routine clinical practice included TDM dosing with a plasma trough concentration target between 15 and 20 mg/L and was based on local guidelines in line with the EUCAST clinical dosing recommendation (see supplementary table 1). During the trial, the in-hospital guidelines changed the target for vancomycin to an AUC_0-24_/MIC target >400 for an MIC of 1 mg/L for unknown pathogens making the target in line with the dosing target for AutoKinetics. In both participating ICUs, the combination of ceftriaxone and ciprofloxacin was the first-choice antibiotic regimen for the treatment of sepsis (of unknown origin) for patients in the trial. Ciprofloxacin was chosen specifically to target gram-negative bacteria. Therefore, the AutoKinetics dosing target for ciprofloxacin was selected to prevent underdosing of septic patients with a gram-negative bacteremia as it is associated with higher incidence of sepsis and consequent mortality [2]. Additionally, previous work from our hospital showed that ciprofloxacin MIC values for suspected pathogens, especially klebsiella species, range up to 1 mg/L [3]. This is disconcerting as klebsiella is among frequently found gram-negative bacteria in ICU culture-positive blood samples in ICU patients [4]. For meropenem, EUCAST maintained a clinical MIC of 2 mg/L while clinical response has been observed for 40% *f* T > MIC [5]. AutoKinetics used an MIC of 1 mg/L based on the low resistance patterns for meropenem in the Netherlands and participating centers [6]. Nonetheless, AutoKinetics maintains a PK safety boundary factor of 4 for the actual dosing target, 100%T>4xMIC, which covers the EUCAST target of time above MIC. For ceftriaxone, EUCAST recommended dosing schedule is 2 grams once daily for pathogens with an MIC up to 1 mg/L and is in line with the AutoKinetics dosing target.

# Study protocol and important changes after trial commencement

The trial was started on February 2^nd^ 2018. After trial initiation three important changes were made to the study protocol and statistical analysis plan.

Firstly, the initial protocol included Cefotaxime as a study antibiotic. Inclusions were never started due to national shortages and changes in the sepsis protocol in one of the participating centres.

Secondly, on April 29^th^ 2018 the inclusion criteria were changed to either elevated serum lactate or vasopressor in any dose rather than both. This change was implemented to account for the clinical workflow in which patients receive fluid resuscitation in the emergency department and initiation of vasopressors. Patients for whom the lactate has normalized due to these interventions were part of the intended study population. We report the number of septic and septic shock patients in the baseline table.

Thirdly, the evaluation of the pharmacometric endpoints has been lowered from 100% to 75% target attainment for both the intervention and control group. Importantly, the dosing targets were not altered and therefore no changes to the study protocol and trial execution were made. The importance of the pharmacometric endpoint change can be illustrated by considering the perfect physician compliance with the dosing advice based on a well-validated PK model. Even under ideal circumstances, some patients would not attain their target. We therefore decided to set the target endpoint to 75% of the target used for calculating dose recommendations. As this represents an arbitrary cut-off point and because any cut-off point will remain a subject of debate, we specified to also explore different cut-off points which will be performed in a secondary analysis.

All changes and a detailed explanation of the rationale behind them have been included in the study protocol that was published on December 19^th^ 2019, before the analysis of any trial data. The study protocols, starting with the originally approved study protocol, version 5, and the updated version 6 with the change in inclusion criteria are available as supplementary files.

# Supplementary material references

1. The European Committee on Antimicrobial Susceptibility Testing. EUCAST Breakpoint tables for interpretation of MICs and zone diameters, version 8.0.; <https://www>.eucast.org/fileadmin/src/media/PDFs/EUCAST_files/Breakpoint_tables/v_8.0_Breakpoint_Tables.pdf (accessed June 28, 2021).
2. Vincent J-L, Mongkolpun W. Current management of Gram-negative septic shock. Curr Opin Infect Dis 2018; 31: 600–5.
3. Van Zanten ARH, Polderman KH, van Geijlswijk IM, van der Meer GYG, Schouten MA, Girbes ARJ. Ciprofloxacin pharmacokinetics in critically ill patients: a prospective cohort study. J Crit Care 2008; 23: 422–30.
4. Sligl W, Taylor G, Brindley PG. Five years of nosocomial Gram-negative bacteremia in a general intensive care unit: epidemiology, antimicrobial susceptibility patterns, and outcomes. Int J Infect Dis 2006; 10: 320–5.
5. Kuti JL. Optimizing antimicrobial pharmacodynamics: a guide for your stewardship program. Revista Médica Clínica Las Condes 2016; 27: 615–24.
6. Bartlett JG, Gilbert DN, Spellberg B. Seven Ways to Preserve the Miracle of Antibiotics. Clin Infect Dis 2013; 56: 1445–50.

# Supplementary Figure 1. AutoKinetics interface


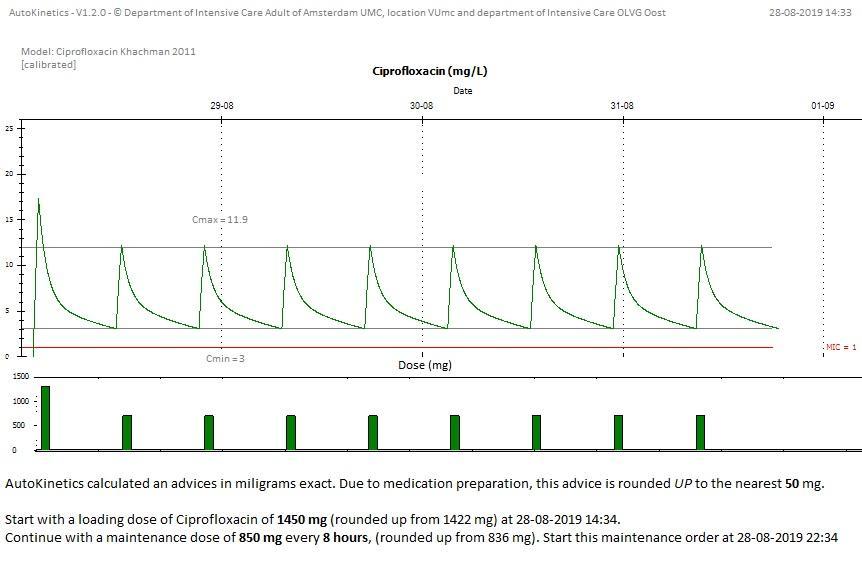


User interface showing the projected antibiotic plasma concentration curve (green line) and a textual dose advice by AutoKinetics (literally translated to English from the original Dutch wording for this supplemental material). The horizontal red line shows the minimal inhibitory concentration used for dosing advice. The horizontal grey lines show the steady state concentration upper and lower limits. AutoKinetics recommends a loading dose as well as a maintenance dose. Dose advice may change over time depending on actual antibiotic doses administered and changes in model parameters.

# Supplementary Figure 2. Dose distribution in the first 24 hours after randomisation


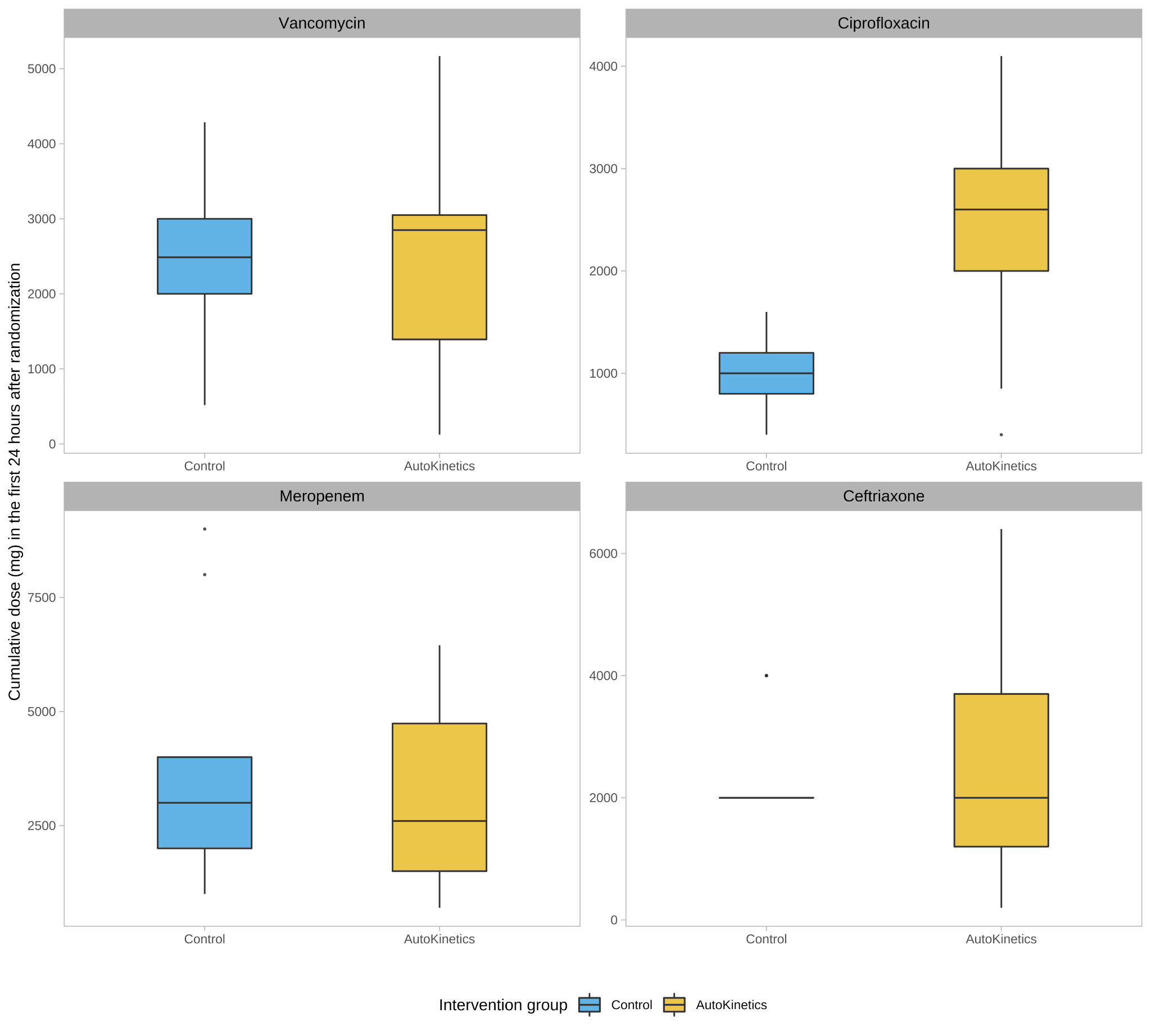


For each antibiotic, median total dose with interquartile ranges are shown for the first 24 hours after randomisation.

# Supplementary Figure 3. Individual dose distribution in the first 7 days after randomisation

**
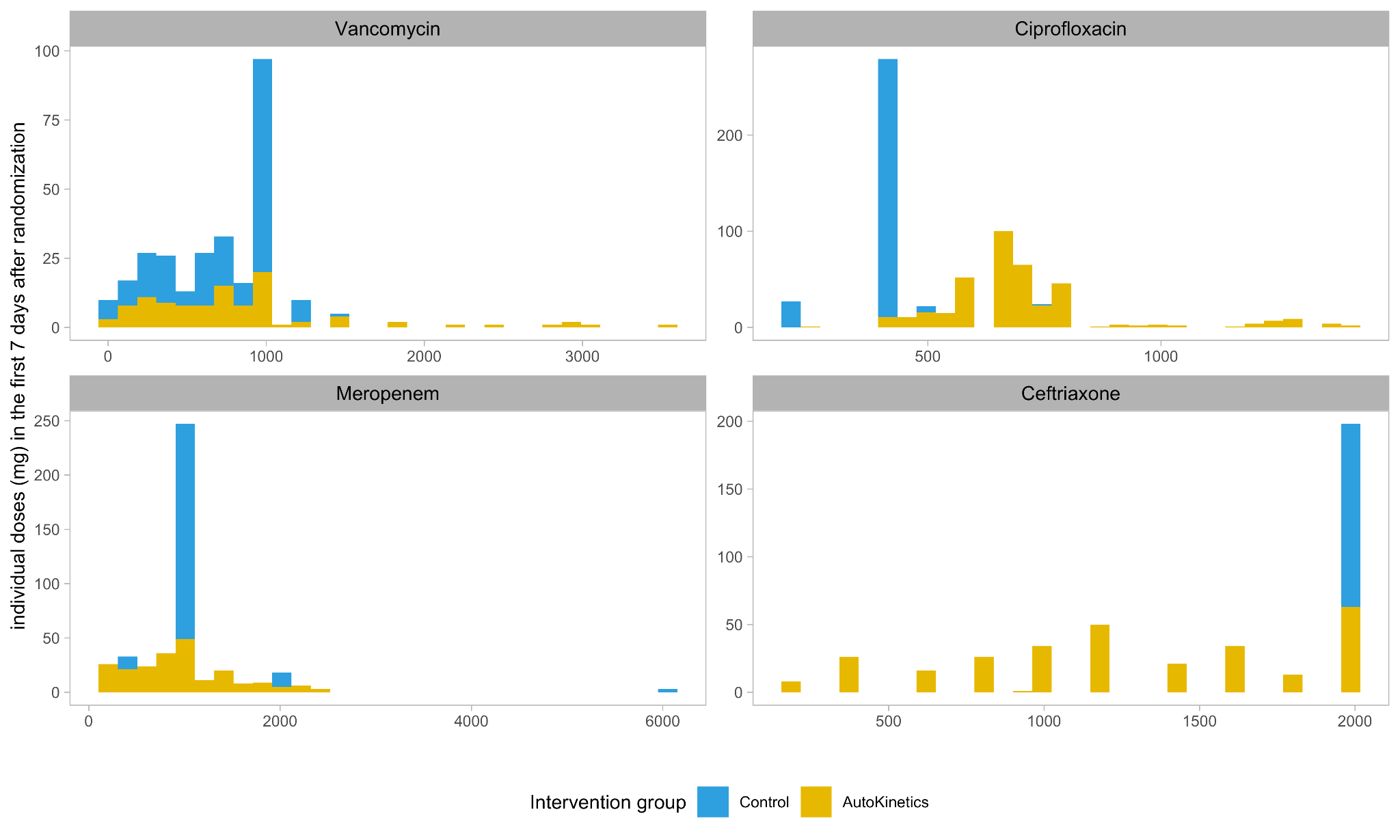
**

For each antibiotic, individual unique doses for each antibiotic are shown up to 7 days after randomisation. For ciprofloxacin, meropenem and ceftriaxone, dose distribution in the control group does not show much variation, while for vancomycin, due to continuous infusion it is more spread out. For vancomycin and ciprofloxacin, some high dosing can be seen. These represent loading doses right after randomisation.

# Supplementary Figure 4. Measured plasma concentration for the primary antibiotic courses


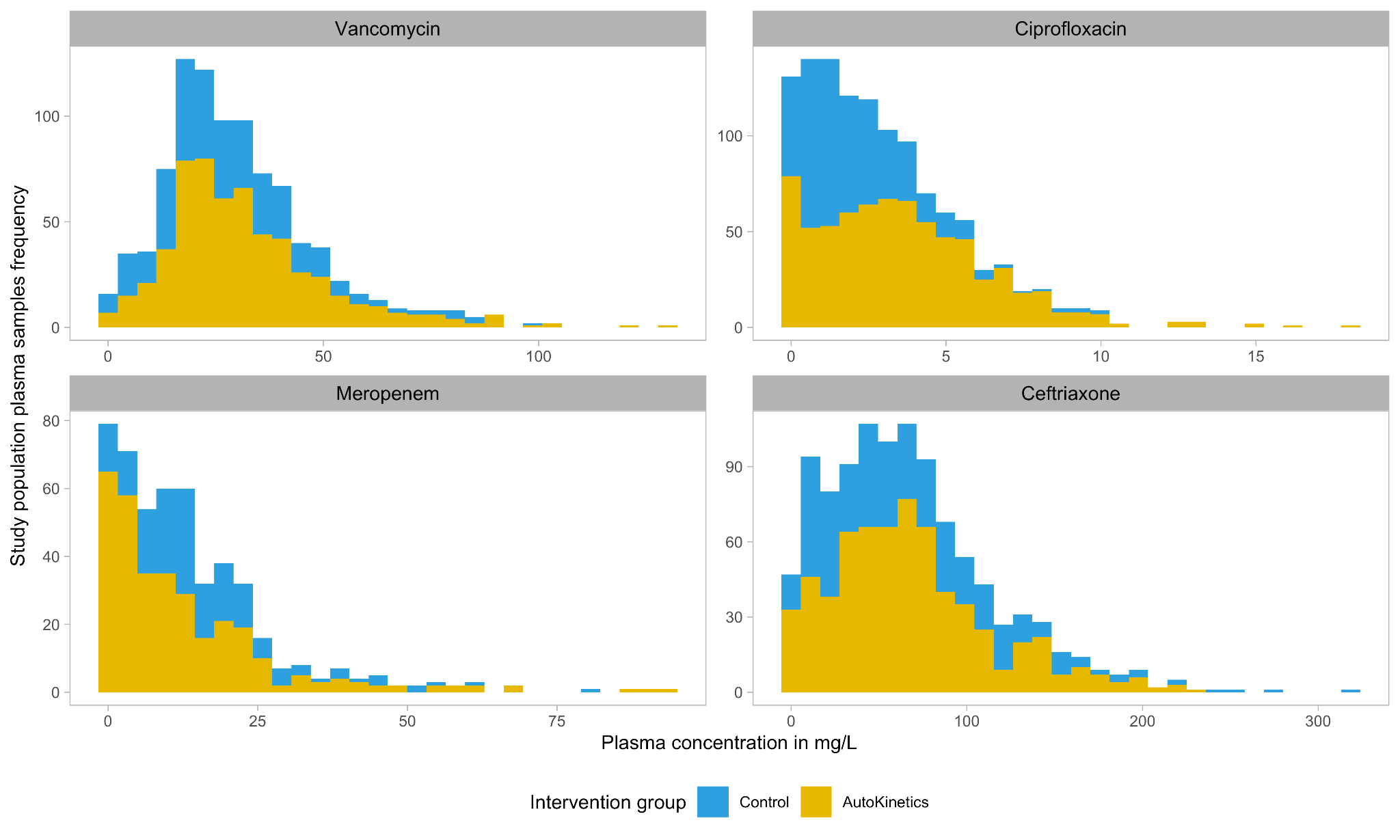


The measured total plasma concentrations – from a mix of trough, peak and interval samples - for the primary antibiotic course are shown in histograms for each antibiotic separately. The Y-axis represents the frequency of measurement and the X-axis represents the measured total plasma concentration.

# Supplementary Figure 5. Probability of target attainment for the first 24 hours after randomisation


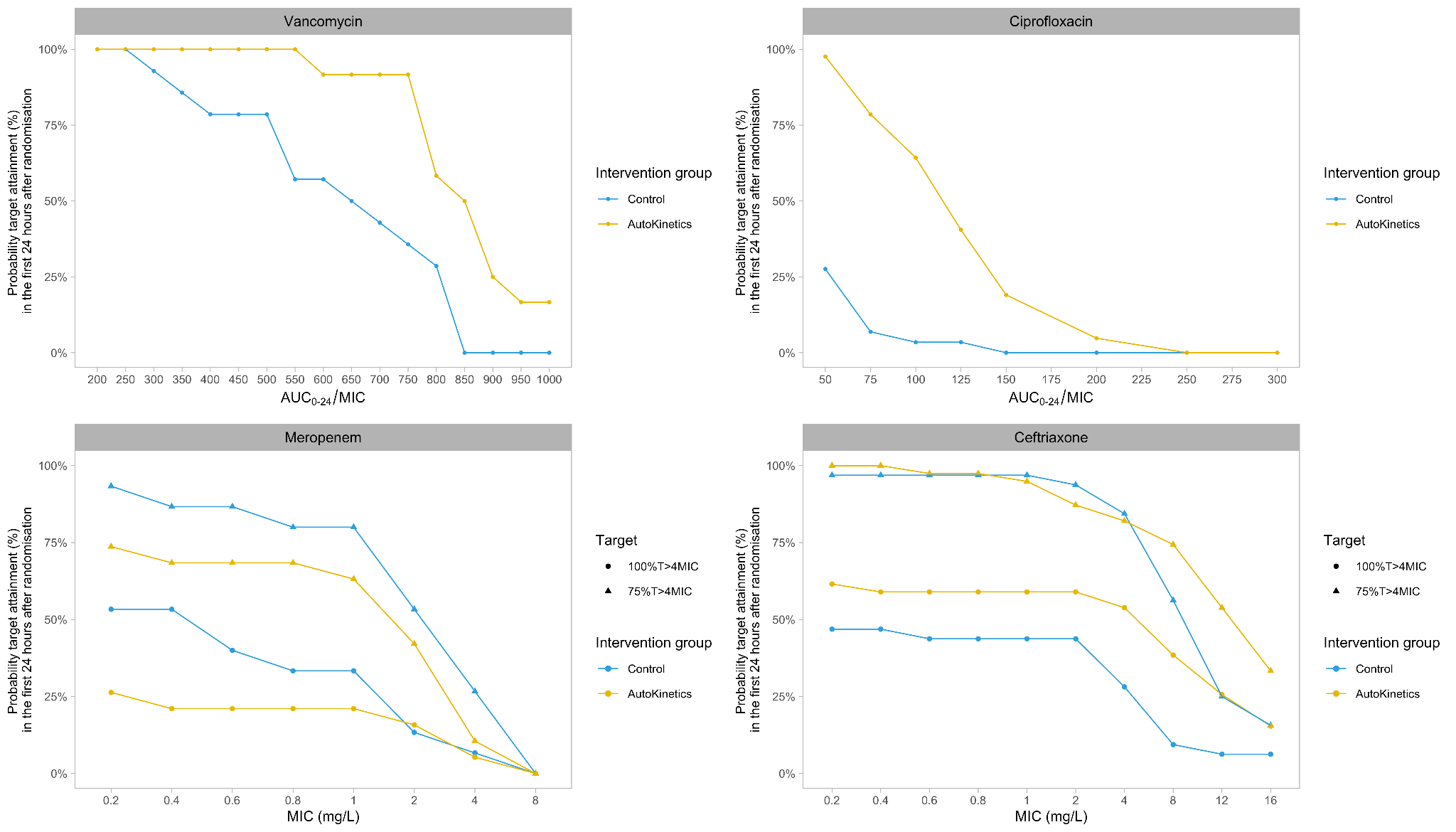


Percentage of primary target attainment (PTA0-24). For vancomycin and ciprofloxacin, PTA0-24 was calculated as the percentage (y-axis) of the target area under the concentration-time curve (AUC) in the first 24 hours divided by the minimum inhibitory concentration (MIC), i.e. AUC0-24/MIC (x-axis) that was reached for vancomycin and ciprofloxacin. For meropenem and ceftriaxone, PTA0-24 was calculated for the primary endpoint as the percentage of time (y-axis) the concentration was equal or greater than 4 times MIC for at least 75% during the first 24 hours. Additionally, PTA0-24 was calculated for the AutoKinetics dosing target of 100% time above 4 times MIC. For meropenem and ceftriaxone we show the target attainment over a range of different MIC values (x-axis).

# Supplementary Figure 6. Average AUC_24_ for the entire antibiotic course versus that after TDM initiation for vancomycin


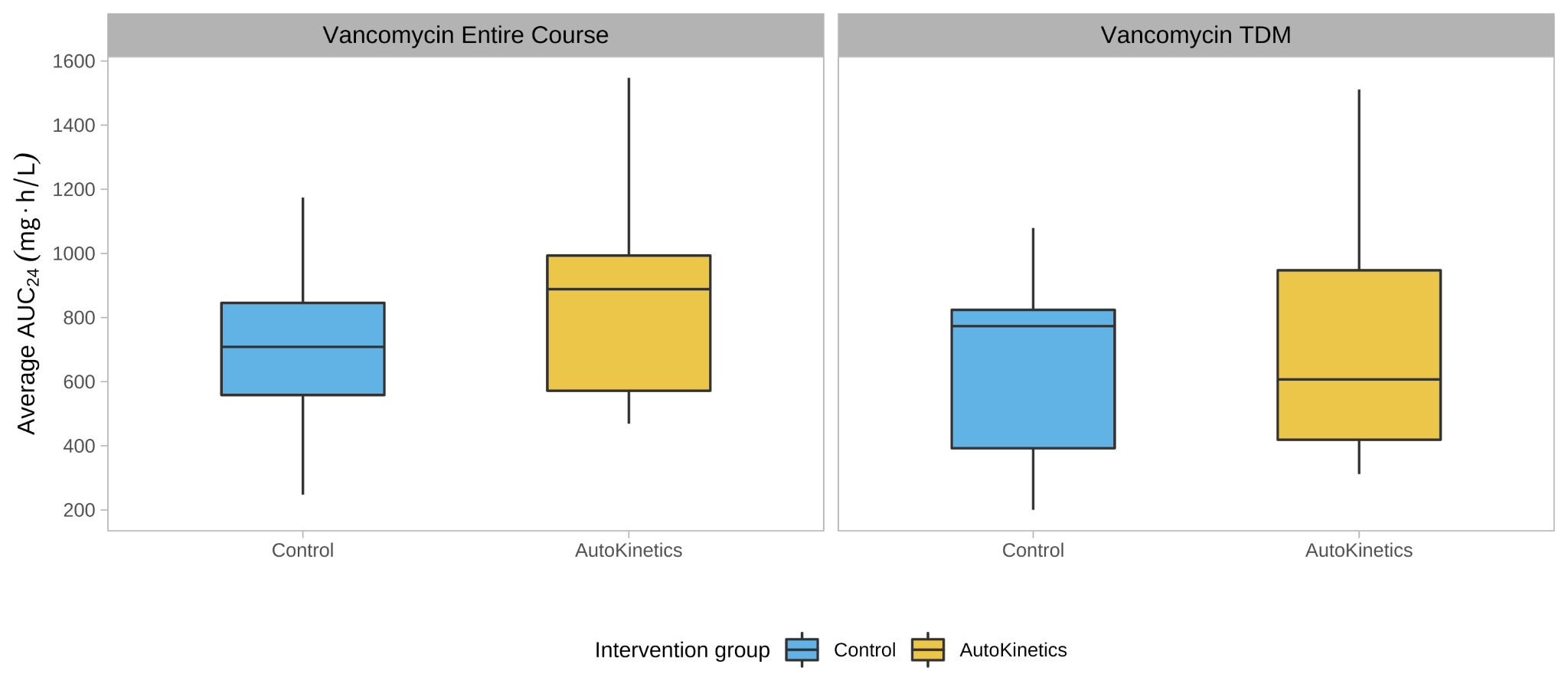


The 24-hour average area under the concentration-time curve is shown for the entire antibiotic course (left), and for the duration of the antibiotic course after antibiotic concentrations for therapeutic drug monitoring (TDM) became available (right). AUC = area under the curve.

# Supplementary Table 1. Antibiotic dosing schedule for routine clinical practice

| **Antibiotic** | **AutoKinetics  Dosing target** | **EUCAST clinical dosing schedule 2018** | **Routine Practice at Amsterdam UMC, location VUmc** | **Routine Practice at OLVG Oost** |
| --- | --- | --- | --- | --- |
| Vancomycin | AUC0-24/MIC>400 | Weight based loading dose + TDM | 1 dd 1000 mg + TDM | 1000mg/24h continuous infusion + TDM |
| Ciprofloxacin | AUC0-24/MIC>125 | 2-3 dd 400 mg | 3 dd 400 mg | 2 dd 400mg |
| Meropenem | 100% T>4xMIC | 3 dd 1000 mg | 3 dd 1000 mg | 3 dd 1000 mg |
| Ceftriaxone | 100% T>4xMIC | 1 dd 2000 mg | 1 dd 2000 mg | 1 dd 2000 mg |
| * Routine practice in Amsterdam UMC, location VUmc for vancomycin included model-based TDM, which was altered over the course of the study. Routine care initially targeted a trough concentration between 15 and 20 mg/L and was changed to target an AUC of 400 mg*h/L using a trough or mid-interval sampling strategy. Plasma sampling for therapeutic drug monitoring was routinely performed after 1-3 days at Amsterdam UMC, location VUmc and after every 24 hours at OLVG hospital. Vancomycin is administered by continuous infusion at OLVG hospital. For meropenem and ciprofloxacin, routine dosing included a dose reduction by 50% and a decreased dosing interval to 2dd if estimated glomerular filtration rate was less than 30 ml/min/1.73m2. | | | | |

# Supplementary Table 2. Prediction error of AutoKinetics pharmacokinetic models

| **Antibiotic** | **Percentage error* (%)** | **Absolute error* (mg/L)** | **Effect on prediction** | **Effect on AutoKinetics dose advice** |
| --- | --- | --- | --- | --- |
| Vancomycin | -2% (-40% to 37%) | -0.4 (-9.3 to 8.4) | no bias | Negligible** |
| Ciprofloxacin | 0% (-37% to 42%) | 0 (-0.6 to 0.8) | no structural bias | Negligible structural bias |
| Meropenem | 120% (-2% to 302%) | 9.1 (-0.1 to 22.1) | overprediction over plasma concentration | At risk for structural underdosing |
| Ceftriaxone | -83% (-97% to -55%) | -38.4 (-60.7 to -16) | underprediction of plasma concentration | At risk for structural overdosing |
| * Presented as median (interquartile range)  ** Effect partially mitigated by application of TDM | | | | |
